# Classification and Regression Tree and Computer Adaptive Testing in Cardiac Rehabilitation: Instrument Validation Study

**DOI:** 10.2196/12509

**Published:** 2020-01-30

**Authors:** Linda Peute, Thom Scheeve, Monique Jaspers

**Affiliations:** 1 Center of Human Factors Engineering of Health Information Technology, Department of Medical Informatics Amsterdam Institute of Public Health Amsterdam University Medical Centers Amsterdam Netherlands; 2 Signal Processing Systems, Video Coding and Architectures Department of Electrical Engineering Eindhoven University of Technology Eindhoven Netherlands

**Keywords:** psychometrics, computing methodologies, mHealth, internet, cardiac rehabilitation, needs assessment

## Abstract

**Background:**

There is a need for shorter-length assessments that capture patient questionnaire data while attaining high data quality without an undue response burden on patients. Computerized adaptive testing (CAT) and classification and regression tree (CART) methods have the potential to meet these needs and can offer attractive options to shorten questionnaire lengths.

**Objective:**

The objective of this study was to test whether CAT or CART was best suited to reduce the number of questionnaire items in multiple domains (eg, anxiety, depression, quality of life, and social support) used for a needs assessment procedure (NAP) within the field of cardiac rehabilitation (CR) without the loss of data quality.

**Methods:**

NAP data of 2837 CR patients from a multicenter Cardiac Rehabilitation Decision Support System (CARDSS) Web-based program was used. Patients used a Web-based portal, MyCARDSS, to provide their data. CAT and CART were assessed based on their performances in shortening the NAP procedure and in terms of sensitivity and specificity.

**Results:**

With CAT and CART, an overall reduction of 36% and 72% of NAP questionnaire length, respectively, was achieved, with a mean sensitivity and specificity of 0.765 and 0.817 for CAT, 0.777 and 0.877 for classification trees, and 0.743 and 0.40 for regression trees, respectively.

**Conclusions:**

Both CAT and CART can be used to shorten the questionnaires of the NAP used within the field of CR. CART, however, showed the best performance, with a twice as large overall decrease in the number of questionnaire items of the NAP compared to CAT and the highest sensitivity and specificity. To our knowledge, our study is the first to assess the differences in performance between CAT and CART for shortening questionnaire lengths. Future research should consider administering varied assessments of patients over time to monitor their progress in multiple domains. For CR professionals, CART integrated with MyCARDSS would provide a feedback loop that informs the rehabilitation progress of their patients by providing real-time patient measurements.

## Introduction

### Background

Given the prominent role of the internet in many patients’ lives nowadays, patient portals are increasingly deployed to involve patients in their care process. These portals, for example, allow fewer time-consuming consultations between patients and health care professionals by integrating batteries of questionnaires needed for diagnosis or as part of a patient’s needs assessment. A precondition of this type of use of patient portals is the high quality of data to be exchanged. This is especially required when the patient portal is linked to a computerized decision support system (CDSS), used by health care providers for advice on therapy planning during the decision-making process. Extensive assessment procedures, however, may result in increased response burden on the patient, possibly resulting in low quality of response data [1-4].

There is a need for assessment procedures of shorter lengths that capture patient questionnaire data while attaining high data quality without undue response burden on patients. Computerized adaptive testing (CAT) methods, using Item Response Theory (IRT), have the potential to meet these needs and offer an attractive option to shorten questionnaires. A CAT algorithm uses information from questions already answered, to select the most appropriate question to be administered next. Therefore, a patient is offered only the fewest possible items. Chien et al [1] verified the effectiveness and efficacy of saving time and reducing the burden on patients through CAT applied on the Activities of Daily Living Scale. They found that mobile nursing services placed at the bedsides of patients could, through a CAT module, reduce the burden on patients and save time more than the traditional paper-and-pencil testing appraisals. Similarly, CAT-based administration of surveys of patient perception substantially reduced patient burden without compromising the precision of measuring patients’ perceptions of hospitalization [2]. Another promising method is classification and regression tree (CART) analysis, originating from clinical decision rules research, which is mostly used to classify patients into clinically important categories [5,6]. It can be used to shorten questionnaires by selecting predictor variables (questionnaire items) that allow different questions to be identified for patients with different levels of complaints of a disease. As an illustration, Lu et al [7] successfully applied CART methods in the development of brief screening tools based on questions from existing psychiatric diagnostic instruments. Potential advantages of both CAT and CART for clinical practice are efficient testing and a reduction in the test burden in patients and, consequently, less measurement error during testing. However, as far as we know, CAT and CART performances in terms of their yield in shortening questionnaire lengths and sensitivity and specificity levels have not been compared.

In this study, we examined if CAT or CART analysis could be used to shorten the questionnaires included in the needs assessment procedure (NAP) of cardiac rehabilitation (CR) patients. CR is a therapy to support patients with cardiac issues in recovering from a cardiac incident in order to improve their physical and physiological condition [8]. To offer a patient a tailored rehabilitation plan, every patient has to complete an NAP including 80-130 questionnaire items of which answer data are sent to a CDSS. We aimed to test which method, CAT or CART, was best suited to reduce the number of questionnaire items in multiple domains (eg, anxiety, depression, quality of life, and social support) used for the NAP, without the loss of data quality.

### Cardiac Rehabilitation in the Netherlands: Case Study

We used the data collected in the Dutch multicenter CARDSS program [8]. Within this program, CR clinics use a CARDSS electronic patient record (EPR) with computerized decision support (CDS) based on the most recent version of the Dutch CR guidelines [9]. The CDS provides CR professionals with advice on a patient-tailored rehabilitation program based on an NAP. The Dutch guideline requires gathering of 80-130 data items regarding a patient’s quality of life, work resumption, psychological and social functioning, and lifestyle. The patient-tailored rehabilitation program can comprise four possible group-based therapies: disease-specific education; exercise training; lifestyle modification; and relaxation and stress management training, supplemented by individual counseling (eg, by a psychologist, dietician, or social worker) and, if needed, different forms of individual therapies. During the NAP, a CR professional can immediately discuss the CDS advice with the patient to set the final patient-tailored rehabilitation plan. To improve the efficiency of the NAP data gathering process, we developed an electronic portal for patients, called MyCARDSS, that patients can use to enter their data, either at home or at the CR clinic. Some patients fill in the NAP in MyCARDSS just before their consultation at the CR clinic, as they need the help of a nurse or do not have an internet connection at home [10]. MyCARDSS is linked to the EPR system.

Data of 2837 CR patients of this multicenter CARDSS Web-based program was included in this study. The CARDSS Web-based dataset comprised patient identification data and CR needs assessment data. We used the database of CARDSS Web-based program to obtain the scores on the individual questions of the patients’ NAP data and the following demographic patient information: age, gender, diagnosis, and cardiac intervention.

### Questionnaires Data

We included data from seven questionnaires used in the NAP for CR, which allow the classification of patients based on the outcome: no (low), mild (moderate), and serious (serious or high) symptoms. The following questionnaires are used in the multicenter CARDSS Web-based program:

The Dutch version of the Quality of Life after Myocardial Infarction (QLMI) is a 27-item questionnaire, scored on a 7-point Likert scale, to measure health-related quality of life for patients after myocardial infarction. It comprises 10 physical dimension items (QLMI-P), subscores ranging from 1 to 7, where subscale scores between 1.0 and 3.39 classify a patient as having a low and >4.0 as having a high exercise capacity; 7 social dimension items (QLMI-S), subscores ranging from 1 to 7, where subscale scores between 1.0 and 4.4 indicate a high, between 4.5 and 5.9 indicate a moderate, and >6.0 indicate a low risk on social dysfunctioning; and 10 emotional dimension items (QLMI-E) [11]. The QLMI-E is not used in the NAP for CR.

The Hospital Anxiety and Depression Scale (HADS) is a 14-item questionnaire, scored on a 4-point Likert scale (0-3), to detect the presence of anxiety and depression. It comprises seven anxiety items (HADS-A) and seven depression items (HADS-D). A total score is not calculated. For both subscales, scores range from 1 to 21. For both HADS-A and HADS-D, subscale scores between 0 and 4 points indicate a low, between 5 and 7 points indicate a moderate, and >8 points indicate a serious risk on anxiety [12].

The Patient Health Questionnaire–9 (PHQ-9) is a 9-item questionnaire on a 4-point Likert scale to detect the presence and severity of mental health disorders with scores ranging from 0 to 27: Scores between 0 and 4 points indicate a low, between 5 and 9 points indicate a moderate, and >10 points indicate a serious risk on depression [13].

The Generalized Anxiety Disorder-7 (GAD-7) is a 7-item questionnaire on a 4-point Likert scale to detect the presence of generalized anxiety disorders. Scores range from 0 to 21: Scores between 0 and 4 points indicate a low, between 5 and 9 points indicate a moderate, and >10 points indicate a serious risk on anxiety [14].

The Multidimensional Perceived Social Support Scale (MPSSS) is a 12-item questionnaire, scored on a 7-point Likert scale, to measure social support and specific availability and satisfaction with support from family, friends, or a special person. Scores range from 12 to 84: Scores between 12 and 64 points indicate a low, between 65 and 78 points indicate a moderate, and >79 points indicate a high level of social support [15].

The Dutch guidelines state that all patients have to fill in the QLMI-P/QLMI-S questionnaire, but CR clinics can choose between a combination of HADS-A/HADS-D or GAD-7/PHQ-9 to assess anxiety and depression levels in their patients. This means that an individual patient fills in the QLMI-P/QLMI-S and HADS-A/HADS-D or QLMI-P/QLMI-S and GAD-7/PHQ-9 questionnaires. Administration of the MPSSS is not mandatory; this questionnaire is used by merely one clinic in the CARDSS Web-based program to assess social support provided to a patient.

## Methods

### Classification and Regression Tree

We first used CART as a method to reduce the number of items of each questionnaire included in the NAP. CART represents a hierarchical model structured as trees for predicting continuous (regression trees) and categorical (classification trees) variables [16,17].

CART models predict a response variable (ie, outcome) based on the values of ≥1 predictors (ie, items within the questionnaire or demographic data) using a technique called recursive partitioning groups [18]. This algorithm looks for subgroups in the dataset in which the response variable is relatively homogeneous. These subgroups become the leaves of the tree [16,18]. At each node in the tree, the recursive partitioning algorithm identifies a predictor variable and a *split* by which cases may be subclassified. This predictor variable and split combination are chosen to have the greatest predictive power among all predictor split combinations at the tree node. Once the cases at the node have been partitioned by the split, the algorithm is applied to both resulting subclassifications. The bottom node of the tree reports a classification for the patient. In most cases, the constructed model is asymmetric, which means that it depends on previous answers on items administered to a patient. For qualitative outcome variables, the resulting tree is a classification tree, and for quantitative outcome variables, it is a regression tree. To shorten the NAP overall, all individual questions of each questionnaire were entered into a CART analysis to develop a CART per questionnaire. The CART analysis selects independent items that differentiate the outcome variable but allows different combinations of the predictor variables in different subgroups, creating flexible questionnaires [16,18]. CART allows the set of questionnaire items presented to a patient to be adapted to the responses already provided by him or her; going left at a node may result in a very different set of questionnaire items being presented as compared with going right. Thus, CART has the potential to shorten the length of the NAP. To determine if CART can shorten the NAP, we determined the mean, maximum, and minimum length of each CART per questionnaire included in the NAP*.*

We also tested if demographic or clinical variables—patient age, gender, cardiac diagnosis, and intervention—as predictors influenced the length of the CART.

### Computer Adaptive Testing

The second method we used to shorten the number of questionnaire items of each questionnaire included in the NAP is CAT. The net result of a CAT is a small, optimal number of items to be administered to the patient without loss of measurement precision. CAT is based on IRT. IRT models are statistical models of the relationship between a person’s score on the construct being measured and the probability of choosing each response on each item measuring that construct. IRT models can be used to evaluate how informative an item is for a specific range of scores and estimate a person’s IRT score [19]. An IRT model expresses a probability (vertical axis) of the selection of each item response category as a function of the score (horizontal axis) on the underlying latent trait (the measured construct, ie, anxiety or depression in this study). To estimate the latent trait, a great number of different IRT models can be used [19]. For questions with ordered response categories, the Graded Response Model (GRM) has been proposed [19]. As the included NAP questionnaires have ordered response options, we fitted a GRM to our data to measure the item parameters of all questionnaire items. A CAT begins with an initial global question; all patients answer the same first item. On the basis of the response to the first item, the score, CI, and latent trait are estimated using maximum likelihood information [19]. The algorithm selects further items based on the highest possible information for the current latent trait score. The latent trait is estimated after each item administration based on the accumulated information combined with the information on the new response [19]. The adaptive testing stops when a stopping rule is met. In this study, CAT stops as soon as a patient can be classified in a low, moderate, or high class with a CI of 95%. A patient had to have answered at least one item before the stopping rule was checked. With this stopping rule, the complete test cycle has a variable length, depending on the patient’s individual responses and the point at which the stopping rule is applied. We did not set a minimum for item administration. Thus, theoretically, the algorithm could stop after the administration of only two or three items, given that the item is informative enough to classify a patient in the low, moderate, or high class. To determine if CAT can shorten the NAP, we determined the mean, maximum, and minimum length of each CAT per questionnaire included in the NAP*.*

### Performance Testing

To determine the performance for both CAT and CART, the sensitivity and specificity were computed. For the regression trees, the root mean squared error (RMSE) and the normalized RMSE (NRMSE) were additionally measured. The RMSE is a measure of the differences (ie, the prediction errors) between values predicted by a regression/classification tree and those actually observed [18]. The RMSE serves to aggregate the prediction errors into a single measure of predictive power. The lower the RMSE or NRMSE, the better the model predicts.

Using the same data for calibration and evaluation of the model results in overly optimistic estimates of performance. Cross-validation is a method for validation of a procedure for model building that avoids the requirement for a new or independent validation set [20]. We, therefore, randomly split the data into two sets: a training set (2127/2837, 74.97%) for calibration of the models and a validation set (710/2837, 25.02%) for evaluating the performance of the CAT and CART. Besides, 10-fold cross-validation was divided into 10 subsets, each subset, in turn, being used to test the performance of the CAT/CART created with the other 9 subsets.

### Software

All analyses were performed in R (R Foundation of Statistical Computing, Vienna, Austria), a programming language for statistical computing. Different packages were used to perform the analyses. R packages for simulating Item Response Theory based on Computerized Adaptive Tests and Latent Trait Modes packages were used for the CAT simulation. R packages for Classification and Regression Training and Recursive Partitioning And Regression Trees were used for CART. The training and test datasets were created with the *caret* package [21,22].

## Results

### Patient Characteristics

Demographic and clinical characteristics of the patients from the clinics participating in the CARDSS Web-based program are shown in Table 1. 

**Table 1:** Demographic and clinical characteristics of 2837 cardiac rehabilitation patients.

Characteristics		Value
**Gender, n (%)**		
	Male	1984 (69.99)
	Female	826 (29.11)
	Missing	27 (0.95)
**Age (years), mean (SD)**		
	Men	65.8 (10.9)
	Women	68.6 (11.3)
	Mean	66.6 (11.1)
**Diagnosis and intervention, n (%)**		
	ACS^a^ (myocardial infarction or unstable angina pectoris) with intervention (CABG^b^, PCI^c^, CABGVALVE^d^, or VALVESUR^e^)	831 (29.29)
	Chronic diagnosis (heart failure or stable angina pectoris)	648 (22.84)
	Elective PCI (PCI without ACS)	604 (21.29)
	Elective CABG (CABG, CABGVALVE, or VALVESUR without ACS)	404 (14.24)
	ACS without intervention	350 (12.33)

^a^ACS: acute coronary syndrome.

^b^CABG: coronary artery bypass grafting.

^c^PCI: percutaneous coronary intervention.

^d^CABGVALVE: coronary artery bypass grafting in combination with heart valve surgery.

^e^VALVESUR: heart valve surgery.

Of the 2837 patients who participated in the program. 69.99% (1984/2837) were male and 29.29% (831/2837) had an acute coronary syndrome; men were younger (mean age 65.8, SD 10.9 years) than women (mean age 68.6, SD 11.3 years).

### Inclusion and Exclusion of Questionnaires

Table 2 provides an overview of the total number of questionnaires fully filled out, missing questionnaires, and insufficiently filled out questionnaires. As explained previously, the Dutch guidelines state that CR clinics can choose between a combination of HADS-A/HADS-D or GAD-7/PHQ-9 to assess anxiety and depression levels in their patients. Administration of the MPSSS is not mandatory; this questionnaire is used by merely one clinic in the CARDSS Web-based program. Patient data on questionnaires were excluded from the CAT and CART analysis if (1) a questionnaire was not filled out by the patient (missing) or (2) a provided questionnaire was insufficiently filled out by a patient to calculate a total score (≥1 item responses missing).

**Table 2:** Number of patients who filled out, did not fill out, or insufficiently filled out the questionnaires (N=2837).

Questionnaire	Fully filled out, n (%)	Missing, n (%)	Insufficiently filled out, n (%)
Quality of Life after Myocardial Infarction - Physical dimension	2633 (92.81)	189 (6.66)	15 (0.52)
Quality of Life after Myocardial Infarction - Social dimension	2633 (92.81)	189 (6.66)	15 (0.52)
Patient Health Questionnaire - 9	1156 (40.75)	213 (7.50)	0 (0.00)
Generalized Anxiety Disorder - 7	1223 (43.11)	244 (8.60)	1 (0.04)
Hospital Anxiety and Depression Scale - Anxiety	1266 (44.62)	152 (5.35)	2 (0.07)
Hospital Anxiety and Depression Scale - Depression	1264 (44.55)	149 (5.25)	4 (0.14)
Multidimensional Perceived Social Support Scale	716 (25.23)	57 (2.00)	5 (0.17)

### Classification and Regression Tree

CART models were built for every questionnaire in the NAP using the training set, resulting in a total of 28 CART models: (1) seven classification trees without the additional clinical/demographic data (ie, age, gender, cardiac diagnosis, and intervention) as features, (2) seven classification trees with the additional clinical/demographic data as features, (3) seven regression trees without the additional clinical/demographic data as features, and (4) seven regression trees with the additional clinical/demographic data as features.

The seven classification and seven regression trees with data on the clinical/demographic variables—age, gender, diagnosis, and intervention—showed no inclusion of these variables in the classification or regression trees.

The developed classification trees comprise four to six levels, with five to eight terminal nodes. The developed regression trees comprise four levels, with five terminal nodes.

### Performances of Computerized Adaptive Testing and Classification and Regression Tree

**Figure 1:**
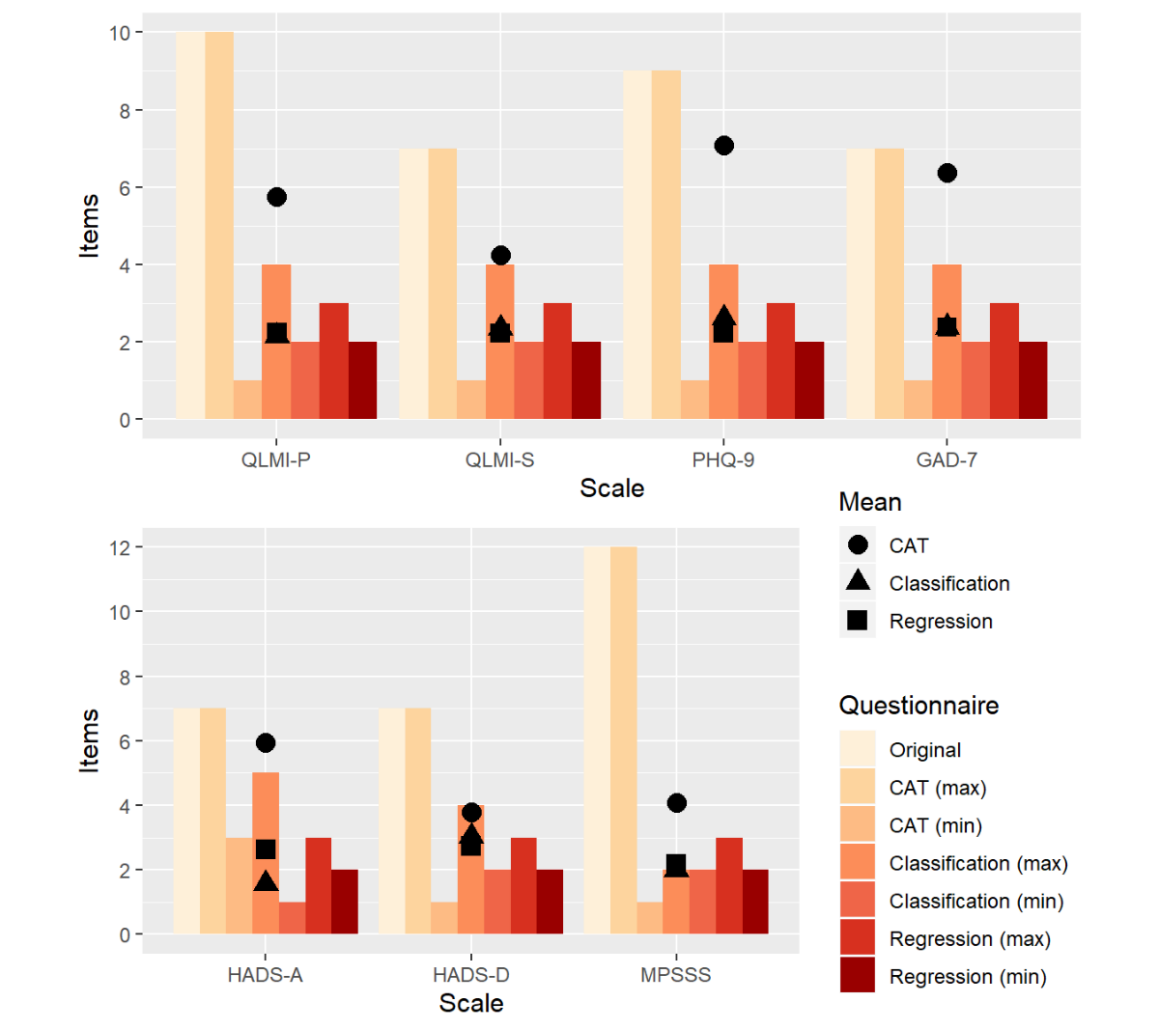
Maximum, minimum, and mean number of items administered in each CAT (computerized adaptive testing) and classification and regression tree. GAD-7: Generalized Anxiety Disorder - 7; HADS-A: Hospital Anxiety and Depression Scale - Anxiety; HADS-D: Hospital Anxiety and Depression Scale - Depression; MPSSS: Multidimensional Perceived Social Support Scale; PHQ-9: Patient Health Questionnaire - 9; QLMI-P: Quality of Life after Myocardial Infarction - Physical dimension; and QLMI-S: Quality of Life after Myocardial Infarction - Social dimension.

**Figure 2:**
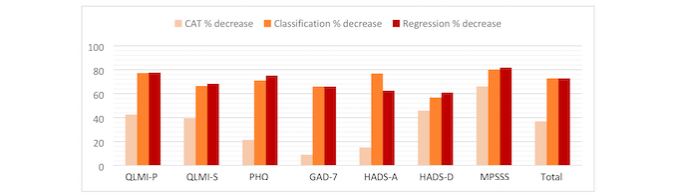
Percentage decrease per questionnaire for computerized adaptive testing and classification and regression tree. QLMI-P: Quality of Life after Myocardial Infarction - Physical dimension; QLMI-S: Quality of Life after Myocardial Infarction - Social dimension; PHQ-9: Patient Health Questionnaire - 9; GAD-7: Generalized Anxiety Disorder - 7; HADS-A: Hospital Anxiety and Depression Scale - Anxiety; HADS-D: Hospital Anxiety and Depression Scale -Depression; MPSSS: Multidimensional Perceived Social Support Scale.

To evaluate the performances of CAT and CART, patient data in the validation dataset were used. Figure 1 shows the maximum, minimum, and mean number of items administered by CART and CAT. Figure 2 displays the percentage decrease per questionnaire for CART and CAT. Table 3 lists the performances of both CAT and CART in terms of sensitivity and specificity per questionnaire. These performance measures are provided for each of the categories—low, moderate, and high—except for the QLMI-P with only low and high classes.

**Table 3:** Sensitivity and specificity of computerized adaptive testing, classification trees, and regression trees by questionnaire (sensitivity and specificity of a class are computed as the class versus the other classes, ie, low versus moderate and high/serious, moderate versus low and high/serious, and high/serious versus low and moderate). High scores on Patient Health Questionnaire - 9, Generalized Anxiety Disorder - 7, Hospital Anxiety and Depression Scales but conversely low scores on Multidimensional Perceived Social Support Scale and Quality of Life after Myocardial Infarction - Physical and Social dimensions instrument indicate that patients report surveyed symptoms.

Questionnaire		Sensitivity			Specificity			Root mean squared error	Normalized root mean squared error
		Low	Moderate	High	Low	Moderate	High		
**Computerized adaptive testing**									
	QLMI-P^a^	0.609^b^	N/A^c^	N/A	1^b^	N/A	N/A	N/A	N/A
	QLMI-S^d^	1	0.008	0.201	0.339	0.691	1	N/A	N/A
	PHQ-9^e^	0.773	0.000	0.988^f^	0.887	0.997	.0581^f^	N/A	N/A
	GAD-7^g^	0.935	0.222	1^f^	0.766	0.996	0.852^f^	N/A	N/A
	HADS-A^h^	0.765	0.333	0.884^f^	0.961	0.887	0.810^f^	N/A	N/A
	HADS-D^i^	0.234	0	1^f^	1	1	0.126^f^	N/A	N/A
	MPSS^j^	1	0	0.515	0.256	1	1	N/A	N/A
**Classification trees**									
	QLMI-P	0.954^b^	N/A	N/A	0.763^b^	N/A	N/A	N/A	N/A
	QLMI-S	0.734	0.735	0.807	0.947	0.786	0.892	N/A	N/A
	PHQ-9	0.907	0.554	0.549^f^	0.852	0.843	0.922^f^	N/A	N/A
	GAD-7	0.931	0.681	0.800^f^	0.869	0.921	0.964^f^	N/A	N/A
	HADS-A	0.941	0.417	0.970^f^	0.969	0.970	0.879^f^	N/A	N/A
	HADS-D	0.979	0.275	0.936^f^	0.938	0.960	0.793^f^	N/A	N/A
	MPSSS	0.644	0.836	0.758	0.970	0.712	0.938	N/A	N/A
**Regression trees**									
	QLMI-P	0.946^b^	N/A	N/A	0.640^b^	N/A	N/A	0.661	0.247
	QLMI-S	0.849	0.648	0.755	0.919	0.787	0.871	0.608	0.184
	PHQ-9	0.861	0.551	0.832^f^	0.833	0.857	0.945^f^	2.696	0.222
	GAD-7	0.957	0.408	0.694^f^	0.645	0.933	0.977^f^	2.231	0.145
	HADS-A	0.750	0.667	0.922^f^	0.966	0.907	0.966^f^	1.833	0.153
	HADS-D	0.640	0.190	0.985^f^	0.996	0.917	0.667^f^	1.582	0.146
	MPSSS	0.489	0.647	0.923	0.977	0.755	0.805	7.962	0.162

^a^QLMI-P: Quality of Life after Myocardial Infarction - Physical dimension.

^b^The quality of Life after Myocardial Infarction - Physical dimension has only a low and high class, so the sensitivity and specificity are computed as low versus high.

^c^Not applicable.

^d^QLMI-S: Quality of Life after Myocardial Infarction - Social dimension.

^e^PHQ-9: Patient Health Questionnaire - 9.

^f^Actual class is serious.

^g^GAD-7: Generalized Anxiety Disorder - 7.

^h^HADS-A: Hospital Anxiety and Depression Scale - Anxiety.

^i^HADS-D: Hospital Anxiety and Depression Scale - Depression.

^j^MPSSS: Multidimensional Perceived Social Support Scale.

#### Classification and Regression Tree

The minimum and maximum number of questionnaire items to be selected for the different questionnaires, based on the training set, are shown in Figure 1. For the classification trees, an average of 2.4 items per questionnaire should be administered (minimum=2 and maximum=3) to classify a patient (Figure 1), with a questionnaire reduction between 60.9% (HADS-D) and 77.6% (QLMI-P), compared with the original questionnaire (Figure 2). For the regression trees, an average of 2.4 items per questionnaire should be administered (minimum=1 and maximum=5) to classify a patient (Figure 1), with a questionnaire reduction between 56.8% (HADS-D) and 77.4% (QLMI-P), compared with the original questionnaire (Figure 2).

For all questionnaires except the HADS-A, the minimum number of questionnaire items (two items) in the classification trees equals those of the regression trees. The maximum number of questionnaire items is higher in the classification (four or five items) than in the regression (three items for all questionnaires) trees, except for the MPSSS (two items) and QLMI-P (three items). The mean number of items and the mean percentage decrease over all questionnaires in the classification trees equal those of the regression trees (2.38). A mean 72% reduction over all questionnaires in the NAP can be realized by CART.

Per questionnaire, the sensitivity and specificity levels of CARTs, classification, and regression trees are displayed in Table 3. The mean sensitivity and specificity of CART over all questionnaires and classes are 0.777 and 0.877, respectively. The mean sensitivity and specificity of regression trees over all questionnaires and classes are 0.743 and 0.840, respectively. The NRMSE of the regression trees ranges from 0.145 (GAD-7) to 0.247 (QLMI-P).

#### Computerized Adaptive Testing

The minimum and maximum number of questionnaire items used by CAT to be selected for the different questionnaires, based on the training set, are shown in Figure 1. With CAT, an average of 5.3 items (minimum=1 and maximum=original length) were needed per questionnaire to classify a patient (our goal was not to classify but to shorten the questionnaire), with a questionnaire reduction between 9.0% (GAD-7) and 45.8% (HADS-D), compared with the original questionnaire (Figure 2). CAT shows a smaller percentage decrease in questionnaire items per questionnaire and a smaller overall decrease, compared with CART (Figure 2). A mean 36% reduction over all questionnaires in the NAP can be realized by CAT. The sensitivity and specificity levels of CATs per questionnaire are displayed in Table 3. The mean sensitivity and specificity of CAT over all questionnaires and classes are 0.765 and 0.817, respectively.

### Computerized Adaptive Testing and Classification and Regression Tree: Questionnaire Comparison

The differences in percentage decrease in questionnaire items per questionnaire between CAT and CART are highest for the PHQ-9 (56.7%), GAD-7 (56.5%), and HADS-A (54.6%). HADS-D (13.0%) and MPSSS (14.9%) show the lowest differences in percentage decrease per questionnaire (Figure 2).

## Discussion

### Principal Findings

This study shows that both CAT and CART can be used to shorten the questionnaires of the NAP used within the field of CR. CART, however, showed the best performance with an overall about twice as large decrease in questionnaire items of the NAP and the highest sensitivity and specificity. Demographic/clinical variables—patient age, gender, cardiac diagnosis, and intervention—as predictors did not influence the length of the CARTs, meaning that these variables do not determine the classification of patients in the trees.

### Relation to Other Studies

CAT has nearly four decades of research behind it but has only been applied more recently to health care. CAT has been used to shorten or develop questionnaires for assessment of fatigue [23], depression [24-26], suicide ideation [4], other mental health disorders [27,28], physical [29] and upper extremity functioning [30], health status in patients with knee osteoarthritis [31], activities of daily living in outpatients with stroke [32], and exposure of nurses to workplace bullying [33,34] and in patient-reported outcome measurement studies [24,29]. Overall, its application has proven to be successful in shortening questionnaires, while patient measurements remained valid and reliable. Some studies even demonstrated that by applying CAT, existing instruments for patient-reported outcomes could be improved. These new instruments reduced the questionnaire burden on patients while increasing measurement precision [29], possibly leading to reduced sample size requirements.

Similarly, half a century has passed since the publication of the first CART algorithms, but again, their application in health care is of a far more recent date.

CART has, for the most part, been used to classify (new) patients into clinically important (risk) categories, such as diabetic nephropathy [35] and colorectal adenocarcinoma [36]. CART has also been applied to define factors associated with delayed treatment of acute myocardial infarction [37] and quality of life [38].

As far as we know, CART has not been used, at least not in the health care domain, with the aim to shorten questionnaires.

To our knowledge, our study is the first to assess the differences in performance between CAT and CART for shortening questionnaire lengths. Overall, CART outperformed CAT, with a larger reduction in the length of the questionnaire for the NAP procedure and in sensitivity and specificity.

Further, we did not observe an influence of the predictors such as age, gender, diagnosis, and intervention in the construction of CARTs. CART would have probably captured interactions across many NAP questionnaire scores and these clinical/demographic variables. Our findings are in concordance with the findings of the study by Miscio et al [39] wherein the inclusion of clinical/demographic data such as age and gender did not have an effect on the construction of CARTs for patient measurement tools.

### Meaning of This Study

Ideally, an NAP procedure for CR including several questionnaires should be highly sensitive and specific, so that few patients with depression/anxiety/social complaints are missed and few without depression/anxiety/social complaints are identified as having complaints. In this context, CART is, overall, more sensitive and specific than CAT, while it shortens the NAP procedure more than CAT. CART analysis has the statistical advantage of being a nonparametric method, with no assumptions about the functional form of the data. CART might further be a good alternative to CAT, as this method not only has the ability to efficiently shorten questionnaires by segmenting patient groups into meaningful subgroups, but it also presents knowledge on these subgroups in a graphical way. These graphs provide a good understanding of how this segmentation was attained. CART, as an algorithmic rather than statistical method, further offers good insight into interactions between variables that are not revealed by linear quantitative research. But CART does not provide distributions, likelihood ratios, or CIs to quantify or support the validity of the findings. For the CATs, we, for example, made use of CIs; we stopped a CAT as soon as a patient could be classified in a low, moderate, or high class with a CI of 95%. For evaluating the performance of both the CAT and CART, we made use of cross-validation by splitting the dataset in a training set for calibration of the models and a validation set. We further applied 10-fold cross-validation on the training and validation sets to validate the generality of the CAT and CARTs.

### Recommendations for Care Practice and Future Research

We demonstrated that an NAP with shortened questionnaires can be used for screening cardiac patients on rehabilitation needs without compromising its measurement accuracy. Both CAT and CART can be used for this purpose, but a CART approach with many questionnaires is rare. Obtaining the information during the joint administration of the shortened questionnaires would take about one-third of the time that it would take with the traditional fixed-length questionnaires, that is, 30 min instead of 90 min per patient. We also plan to extend the MyCARDSS portal with adaptive tests that will be administered to our CR patients over time, at their home or just before their consultation at the clinic. The results of these assessments will be interfaced with the CARDSS EPR, which is easily accessible by the CR professionals from any device. For CR professionals, CART integrated with MyCARDSS and CARDSS would then provide a feedback loop that informs the rehabilitation progress of their patients by providing these real-time patient measurements. An example for future research is to monitor our CR patients’ physical and mental health progress by these varied assessments. Although dimension reduction strategies have been employed for numerous data problems, they are scantly discussed in the context of analyzing survey data. Another example for future research, thus, is to examine the performance of other methods such as dimensionality reduction techniques, for example, principal component analyses (PCA) for reducing questionnaire lengths. Dimension reduction techniques can be an effective approach for reducing dimensionality in more complex survey data sources than the data source used in this study. Methods such as CART, CAT, and PCA could, for example, be applied on federal and other publicly available datasets to further improve the validity and generalizability of the findings of this case study and conduct more efficient and cost-effective surveys in the future.

### Strengths and Limitations

A strength of this study pertains to its large sample size; 2837 patients from various CR clinics completed the various NAP questionnaires, confirming the external validity of the findings. The number of fully filled out questionnaires per type of questionnaire varied from 716 to 2633, with MPSSS being used in one CR clinic and having the lowest response rate. The original version of the MPSSS, furthermore, has a low number of questionnaire items in comparison with QLMI, for example. This might have impacted the CAT and CART analysis. It, hence, remains uncertain whether the results, particularly those of MPSSS, can be generalized to other CR clinics. An even larger sample size could have led to a more precise estimation of NAP questionnaire lengths needed to determine patients’ specific CR needs.

Another strength of this study is the application, exploration, and comparison of the two techniques for shortening questionnaires. The use of CART analysis as one of these techniques has been suboptimal at the least; we did not find any study using CART aiming at reducing questionnaire lengths. Finally, the Dutch guideline for CR prescribes that CR clinics can choose between a combination of HADS-A/HADS-D or GAD-7/PHQ-9 to assess anxiety and depression levels in patients. We do not know if these combinations of questionnaires are equally valid in measuring the constructs of anxiety and depression. With CART, a combination of HADS-A/HADS-D would result in a reduction in questionnaire lengths of six and five items, respectively, whereas the combined GAD-7/PHQ-9 would lead to a reduction of seven and five items, respectively.

Finally, we did not examine if the questionnaires of the NAP produced similar results when patients filled them in on the Web through MyCARDSS at their home or in the CR clinic.

### Conclusions

CART and CAT both have shown to be accurate methods for reducing the length of the NAP used in the field of CR. Of both methods, CART overall showed the largest decrease in the number of questionnaire items and the best performance in terms of sensitivity and specificity. This study is the first to apply and compare the performances of CAT and CART for shortening questionnaires, and it demonstrated that the use of CART analysis would be a step forward in the development of a shorter NAP questionnaire for CR patients and possibly other questionnaires.

Instead of using long, fixed-length questionnaires on paper or on the Web for the NAP, a much smaller set of questionnaire items will suffice to identify patients’ varying needs for CR, without the loss of information and data quality and an excessive burden on the patient.

## Abbreviations

CART: classification and regression tree
CAT: computerized adaptive testing
CDS: computerized decision support
CDSS: computerized decision support system
CR: cardiac rehabilitation
EPR: electronic patient record
GAD-7: Generalized Anxiety Disorder - 7
GRM: Graded Response Model
HADS-A: Hospital Anxiety and Depression Scale - Anxiety
HADS-D: Hospital Anxiety and Depression Scale - Depression
IRT: Item Response Theory
MPSSS: Multidimensional Perceived Social Support
NAP: needs assessment procedure
NRMSE: normalized root mean squared error
PCA: principal component analyses
PHQ-9: Patient Health Questionnaire - 9
QLMI-E: Quality of Life after Myocardial Infarction - Emotional dimension
QLMI-P: Quality of Life after Myocardial Infarction - Physical dimension
QLMI-S: Quality of Life after Myocardial Infarction - Social dimension
RMSE: root mean squared error
